# Temporal Learning and Rhythmic Responding: No Reduction in the Proportion Easy Effect with Variable Response-Stimulus Intervals

**DOI:** 10.3389/fpsyg.2016.00634

**Published:** 2016-05-02

**Authors:** James R. Schmidt

**Affiliations:** Department of Experimental Clinical and Health Psychology, Ghent UniversityGhent, Belgium

**Keywords:** proportion easy, proportion congruent, temporal learning, response-stimulus intervals, rhythmic responding, timing

## Abstract

The present report further investigates the proportion easy effect, a conflict-free version of the proportion congruent effect. In the proportion easy paradigm, it is observed that the difference in performance between easy (high contrast) and hard (low contrast) items is smaller in a task with mostly hard items relative to a task with mostly easy items. This effect has been interpreted as evidence for temporal learning: participants learn a faster pace (i.e., rhythm of responding) in the mostly easy context, which boosts the contrast effect, and a slower pace in the mostly hard context. In the present experiment, intervals between trials were either fixed or randomly varied from trial to trial. Interestingly, the proportion easy effect was still present with variable intervals. These data suggest that participants do not learn the regularity in timing from one response to the next (which was highly inconsistent with variable intervals). As one alternative, participants might be learning the intervals between stimulus onset and responses, which were not manipulated. They could then use this learned timing information to prepare for responding at the anticipated time, resulting in rhythmic responding. The results further imply that variable response-stimulus intervals are insufficient for controlling for rhythmic biases.

## Introduction

Often when meaning to study cognitive processes that are responsive to the informational content of stimuli, researchers are accidentally studying rhythmic biases (e.g., see Lupker et al., 1997; Grosjean et al., 2001). For instance, consider the *proportion congruent effect* (Lowe and Mitterer, 1982). In the Stroop task (Stroop, 1935), participants respond to the print color of color words, and performance is worse on incongruent trials (e.g., the word “green” in red) relative to congruent trials (e.g., “red” in red). This congruency effect is smaller when trials are mostly incongruent (e.g., 75% incongruent, 25% congruent) relative to when trials are mostly congruent (e.g., 75% congruent, 25% incongruent). The typical account of this proportion congruent effect is in terms of conflict adaptation (e.g., Cohen et al., 1990; Lindsay and Jacoby, 1994; Botvinick et al., 2001). That is, it is argued that when conflict is frequent, attentional control is increased, leading to a diminished effect of the word on performance. In other words, informational conflict between the word and color leads to adjustments in attention.

However, there are numerous problems with the conflict adaptation view (Schmidt and Besner, 2008; Grinband et al., 2011; Atalay and Misirlisoy, 2012, 2014; Grandjean et al., 2013; Schmidt, 2013a,c, 2014a, in press; Hazeltine and Mordkoff, 2014; Schmidt et al., 2015). Most relevant for the present report, the temporal learning account (Schmidt, 2013b; see also, Kinoshita et al., 2011) suggests that the proportion congruent effect is due (in part) to differences in the pace (or rhythm) of the mostly congruent and mostly incongruent conditions, and not due to the informational conflict *per se*. In particular, the congruency effect in the mostly congruent condition is increased due to a fast rhythm: participants anticipate responding early in a trial (i.e., due to the high frequency of easy congruent trials), and responding is speeded somewhat if they are able to respond when they have anticipated being able to. This *temporal expectancy benefit* typically occurs on congruent trials, where participants are able to respond at the anticipated time (i.e., when the rhythm can be maintained). However, on incongruent trials participants simply do not have enough evidence for the correct response at the predicted time. The rhythm is broken and responding is slowed. Thus, the congruency effect is increased.

In the mostly incongruent condition, it is the reverse: participants expect to respond later in the trial (i.e., due to the high frequency of hard incongruent trials), making them highly prepared for an incongruent response. Because they do not anticipate a response earlier in the trial, they are less prepared to respond as quickly as they could to a (less frequent) congruent trial. In this way, the proportion congruent effect might be due to differences in the pace of the mostly congruent and mostly incongruent conditions. The informational content of the trials (e.g., conflict vs. non-conflict) may therefore be only very indirectly related to the effect. The goal of the present report is not to challenge the conflict adaptation view, but to better understand the rhythmic responding biases that might contribute to the effect.

**FIGURE 1:**
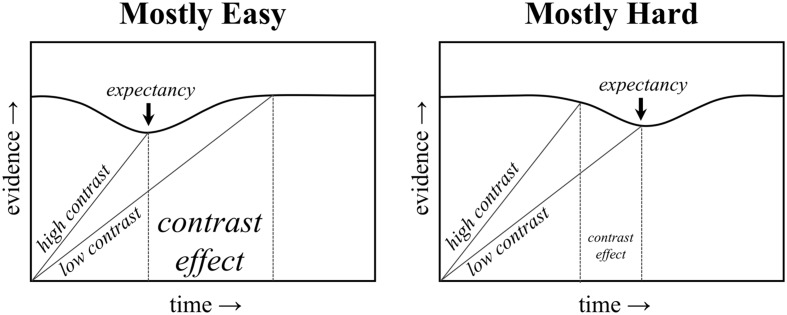
**Illustration of a temporal expectancy mechanism.** The threshold for responding decreases temporarily at the expected time. An earlier expectancy benefits high contrast trials in the mostly easy condition, and a later expectancy benefits low contrast trials in the mostly hard condition.

In this vein, recent studies by Schmidt (2013b, 2014b; Schmidt et al., 2014) provide a useful way for studying rhythmic biases more directly. In particular, a “proportion congruent”-like effect can still be observed with non-conflicting stimuli. In the *proportion easy* task, participants are simply presented with target letters (i.e., no distracters) that are either easy or hard to see (i.e., high or low contrast with the background). The proportion of easy to hard items is then manipulated. Similar to a proportion congruent effect, the difference between easy and hard items (i.e., the stimulus contrast effect) is smaller in the mostly hard condition relative to the mostly easy condition. An illustration of how the above-described temporal learning mechanism can explain the proportion easy effect is presented in **Figure 1**. Of course, the proportion easy effect cannot be due to conflict adaptation, given the absence of conflict in the task (i.e., there is no distracting stimulus to compete with the target letter). Temporal learning can explain both effects, however, suggesting that the proportion congruent effect might be a “proportion easy” effect in disguise.

**FIGURE 2:**
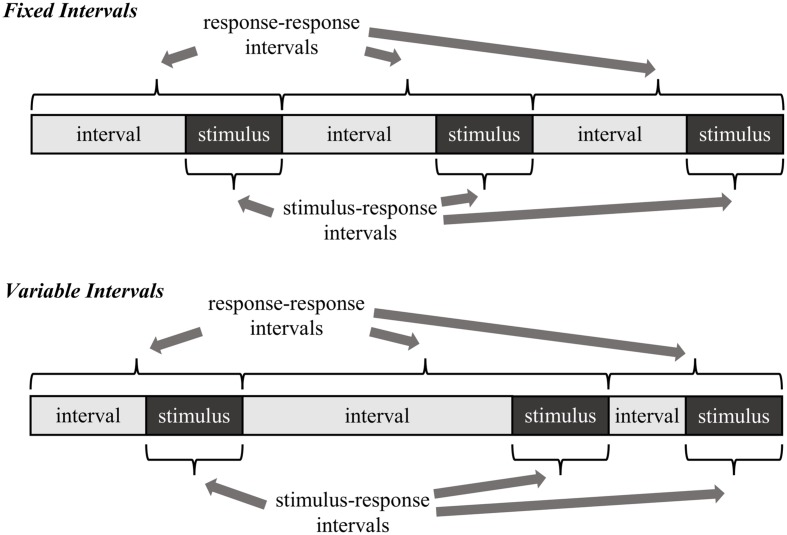
**Two example temporal learning mechanisms as they relate to fixed versus variable intervals.** Note that with variable intervals, the stimulus–response intervals can still remain regular, whereas the response–response intervals cannot.

The goal of the present research is twofold. Preceding with the assumption that the proportion easy effect *is* a rhythmic-based effect, the first goal is to determine what temporal regularities participants are actually learning. Two alternatives are considered, both illustrated in **Figure 2**. The first possibility, called here the *stimulus–response learning account*, is that participants learn the interval between stimulus onset and the response (i.e., the response time). In other words, participants learn to expect a response relative to the moment a stimulus is encountered. The second possibility, called here the *response–response learning account*, is that participants learn the interval between one response and the next (i.e., the intertrial interval). In other words, participants learn to expect a response relative to the time they made their last response.

In order to distinguish between the stimulus–response and response–response learning accounts, the regularity of the response-stimulus interval (RSI) is manipulated in different blocks of trials. If it is the case that participants learn the timing between one response and the next, then randomly varying the RSI should impair rhythmic timing (and therefore the proportion easy effect). That is, there is no longer a consistent response–response rhythm to learn, also illustrated in **Figure 2**. Indeed, proportion easy (in addition to proportion congruent and congruency sequence) effects have been shown to be strongly influenced by the timing of the immediately preceding trial. If response–response intervals are being learned, then there will rarely be a close match between the previous and current trial with variable intervals. In contrast, there will by definition be a much closer match with fixed intervals. Of course, one might propose that participants could still learn the *average* response–response timing in the variable interval condition, but most trials will violate this average. Thus, at minimum, the proportion easy effect should be considerably disrupted with variable intervals if response–response intervals are learned. On the other hand, if participants learn the intervals between stimulus onset and responding, then variations in the RSI should have relatively minimal effect on rhythmic responding (and therefore the proportion easy effect): stimulus–response intervals (i.e., response times) can still be regular, even if the RSI is not.

It is known that varying RSIs does slow performance (Grosjean et al., 2001), but this could be due to one or more of several processes. Of course, the response–response learning account suggests that overall responding is slowed due to an impairment in rhythmic responding. Alternatively, fixed timing of RSIs might aid preparation for stimulus processing (Jones, 1976; Ellis and Jones, 2010). That is, participants might anticipate when to *attend* for a stimulus, allowing for quicker sampling of the target when its onset can be successfully predicted (Laming, 1979a,b). This temporal attending account does predict an overall slowing of responses when stimulus onset is uncertain, but does not necessarily predict an impairment of rhythmic response biases (e.g., if the stimulus–response intervals are learned). As such, the proportion easy effect might still be observed.

The second aim of the present research is interrelated with the first. Determining whether an observed behavioral result is due to the informational content of stimuli or a simple rhythmic bias can be very difficult to disentangle. For instance, if one wishes to determine whether conflict adaptation plays a role in the proportion congruent effect, then it is necessary to rule out rhythmic biases. That is, if a rhythmic responding bias can produce a proportion congruent effect on its own, then it is uncertain whether conflict adaptation must additionally be assumed to explain the effect. If conflict adaptation *does* play a role in the effect, then the effect should not be eliminated by controlling for rhythmic biases. This is challenging, however, because increasing or decreasing the proportion of congruent relative to incongruent trials will *inherently* adjust the rhythm of the task. One can use previous trial RT as a proxy for rhythmic pace. Consistent with the temporal learning view, the congruency effect is larger the faster the response time was on the previous trial and controlling for previous trial RT reduces the proportion congruent effect (Kinoshita et al., 2011; Schmidt, 2013b). However, we cannot simply interpret a remaining proportion congruent effect as conflict adaptation. A statistical control for previous trial RT is not sufficient to fully rule out rhythmic biases, because the rhythm is likely set by more than just the immediately preceding trial.

Given the above difficulties, how can a researcher sufficiently eliminate rhythmic biases from a design? Here, the proportion congruent effect is used as a specific example, but these concerns are broadly applicable to any design in which a difference in task rhythm is present for two conditions: larger effects will be observed with a faster rhythm than with a slower one. If it is the case the participants learn response–response intervals (i.e., rather than stimulus–response intervals), then randomly varying RSIs might prove sufficient to eliminate rhythmic biases. Thus, though the focus is primarily to determine the mechanism underlying rhythmic biases in the proportion easy paradigm, the present experiment also provides a test for one potential way of dissociating rhythmic and informational biases in the proportion congruent paradigm.

## Materials and Methods

The experiment tests whether predictability in the timing of events plays a role in rhythmic responding. To achieve this, the proportion easy paradigm is used. Participants performed both mostly easy and mostly hard blocks. For half of the blocks, the RSI between trials remained fixed at 600 ms (fixed condition). For the other half of the blocks, the RSI varied randomly from trial to trial (variable condition). Two predictions follow from the response–response variant of the temporal learning perspective. First, response times should be overall slower in the variable condition, because the time that the stimulus appears is unpredictable (Granjon et al., 1973; Requin et al., 1973). Second and more critically, the proportion easy effect should be disrupted in the variable condition. In contrast, the stimulus–response variant of the temporal learning account does not make the latter prediction: an effect should be observed in both the fixed and variable interval conditions, and there is no clear *a priori* reason to expect a larger effect in one condition over the other.

### Participants

Twenty-one undergraduates of Ghent University participated in exchange for €5. The study was approved by the Ethics Committee at Ghent University.

### Apparatus

Stimulus and response timing were controlled by E-Prime 2 software (Psychology Software Tools, Pittsburgh, PA, USA). Responses were made on a laptop PC AZERTY keyboard by pressing the D, F, J, and K keys for the letters D, F, J, and K, respectively.

### Design

Stimuli consisted of the letters D, F, J, and K presented in high contrast whitish gray (200, 200, 200) and low contrast darker gray (110, 110, 100), representing high and low contrast items, respectively. All stimuli were presented in bold 18 pt Courier New font. There were four blocks of trials, each with 200 trials (800 total). In two blocks, trials were mostly easy. In these blocks, letters were presented 70% of the time in high contrast and 30% in low contrast. The remaining two blocks were mostly hard. In these blocks, letters were 30% high contrast and 70% low contrast. Orthogonal to this, half of the blocks had a fixed RSI, and half had variable RSIs. Four counterbalancing orders were run: (1) fixed mostly easy, fixed mostly hard, variable mostly easy, variable mostly hard, (2) variable mostly easy, variable mostly hard, fixed mostly easy, fixed mostly hard, (3) fixed mostly hard, fixed mostly easy, variable mostly hard, variable mostly easy, and (4) variable mostly hard, variable mostly easy, fixed mostly hard, fixed mostly easy. All trials were selected at random with replacement.

### Procedure

All stimuli were presented on a medium gray screen (100, 100, 100), which was only slightly different from the low contrast color and very different from the high contrast color. In the dimly lit testing room, both types easily pop out on the screen, but the latter are faster to identify. Each trial began with a blank screen for 200 ms, followed by a fixation “+” for 100 ms, followed by another blank screen. In fixed interval blocks, this blank screen was presented for 300 ms on all trials. In variable interval blocks, this blank screen randomly varied between 0 and 600 ms on a trial-by-trial basis. Thus, the total RSI was either fixed at 600 ms or varied between 300 and 900 ms with a rectangular (continuous uniform) distribution. Note that this means that the average RSI in both conditions is the same (600 ms), but that the RSI in the variable interval condition *changes*, on average, by about 200 ms (in either direction) from one trial to the next (*SD* = 140 ms; range: 0 – 600 ms). This was followed by the target letter until either (a) a response was made or (b) 2000 ms elapsed without a response. The next trial began immediately if the response was correct. If a participant responded incorrectly or failed to respond in 2000 ms, “XXX” in red (255, 0, 0) was presented for 500 ms.

### Data Analysis

Correct response times and percentage errors were analyzed. Trials on which participants failed to respond in 2000 ms were eliminated from the analysis (<0.3% of trials). All participants had sufficiently good accuracy (>80%), so no participants were excluded. Trials following an error were not removed from the analysis, but follow-up analyses confirmed that adding this trim had no influence on the results reported below. Because the order of the four blocks varied from one participant to the next, it might have been possible that block order effects influenced the observed results. As such, initial analyses were performed with the counterbalancing factor included. However, this revealed no confounding influence on the results. All significant findings remained significant, and all non-significant findings remained non-significant. More critically, counterbalancing did not influence the proportion easy effect or the (null) interaction between RSI condition and the proportion easy effect. For brevity, the analysis without the counterbalancing order is reported.

## Results

### Response Times

**FIGURE 3:**
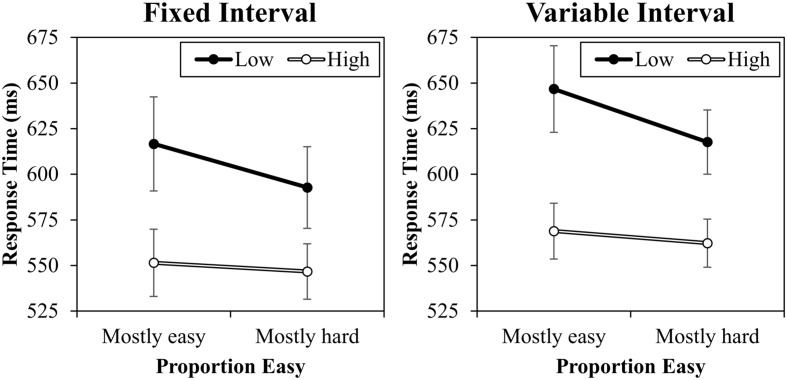
**Response times in milliseconds (with standard errors) for fixed and variable intervals**.

The response time data are presented in **Figure 3**. A contrast (high vs. low) by proportion easy (mostly easy vs. mostly hard) by interval type (fixed vs. variable) ANOVA was conducted. This revealed a significant main effect of contrast, *F*(1,20) = 24.567, *MSE* = 6388, *p* < 0.001, $\eta_{p}^{2}$ = 0.55, because high contrast trials were responded to faster (557 ms) than low contrast trials (618 ms). There was also a main effect of interval, *F*(1,20) = 7.895, *MSE* = 2562, *p* = 0.011, $\eta_{p}^{2}$ = 0.28, because responding was overall slower in the variable interval condition (599 ms) than in the fixed interval condition (577 ms). The main effect of proportion easy was marginal, *F*(1,20) = 4.270, *MSE* = 2547, *p* = 0.052, $\eta_{p}^{2}$ = 0.18, because average RT was slower in the mostly easy condition (596 ms) relative to the mostly hard condition (580 ms). Critically, proportion easy and contrast interacted, *F*(1,20) = 13.819, *MSE* = 329, *p* = 0.001, $\eta_{p}^{2}$ = 0.41, indicating that the contrast effect was larger in the mostly easy condition (72 ms) than in the mostly hard condition (51 ms). It is noteworthy that this effect was primarily driven by changes in low contrast trials: low contrast trials were significantly faster in the mostly hard condition (605 ms) than in the mostly easy condition (632 ms), *F*(1,20) = 9.357, *MSE* = 1562, *p* = 0.006, $\eta_{p}^{2}$ = 0.32. This is as expected (e.g., Schmidt, 2014a). There was no significant difference between high contrast trials in the mostly easy (560 ms) and mostly hard conditions (554 ms), *F*(1,20) = 0.525, *MSE* = 1317, *p* = 0.477, $\eta_{p}^{2}$ = 0.03. It is worth noting that while the overall proportion easy effect is robust in this paradigm, these finer comparisons on easy and hard items vary from study to study in both previously published reports (cf., Schmidt, 2013b, 2014b) and unreported data from our lab, with the effect sometimes appearing in the easy items, sometimes in the hard items, and sometimes symmetrically in both, even with identical designs. This is probably due to the fact that proportion easy is manipulated between blocks, which introduces noise into these finer comparisons. Also interesting, there was no three-way interaction, *F*(1,20) = 0.090, *MSE* = 335, *p* = 0.767, $\eta_{p}^{2}$ < 0.01. Indeed, the interaction was even (slightly) in the opposite direction that the response–response learning account would predict numerically (-3 ms). Because this non-significant effect might represent a true (or near true) null or merely a Type 2 error, a Bayes factor was calculated using the calculator of Dienes (2014). For this, the originally reported (Schmidt, 2013b) 38 ms estimate of the proportion easy effect (and therefore possible change in the proportion easy effect) was used as the maximum bound and 0 ms as the minimum bound with a uniform distribution and the sample mean (interaction) of -3.3637 (*SE*: 11.3017). The resulting Bayes factor for the interaction was 0.30. Because this value is less than 1/3 (0.33), this represents strong evidence for the null hypothesis, supporting the notion that variable RSIs have minimal effect on the proportion easy effect. No other effects were significant (*F*s < 2.620, *p*s > 0.121). Supplementary analyses indicate that the proportion easy interaction was significant both in the fixed interval condition (19 ms), *F*(1,20) = 9.729, *MSE* = 197, *p* = 0.005, $\eta_{p}^{2}$ = 0.33, and in the variable interval condition (23 ms), *F*(1,20) = 5.694, *MSE* = 467, *p* = 0.027, $\eta_{p}^{2}$ = 0.22.

### Percentage Errors

**FIGURE 4:**
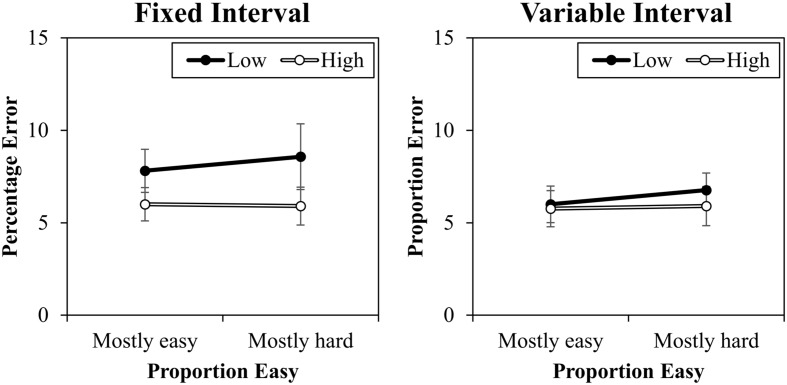
Percentages errors (with standard errors) for fixed and variable intervals.

The percentage error data are presented in **Figure 4**. A contrast (high vs. low) by proportion easy (mostly easy vs. mostly hard) by interval type (fixed vs. variable) ANOVA was conducted. This revealed a significant main effect of contrast, *F*(1,20) = 6.709, *MSE* = 12, *p* = 0.017, $\eta_{p}^{2}$ = 0.25, because there were less errors to high contrast trials (5.9%) than to low contrast trials (7.3%). There was also a marginal interaction between interval and contrast, *F*(1,20) = 3.696, *MSE* = 8, *p* = 0.069, $\eta_{p}^{2}$ = 0.16, because there was a slightly larger contrast effect in the fixed interval condition (2.2%) than in the variable interval condition (0.5%). Proportion easy and contrast did not interact in errors, *F*(1,20) = 0.047, *MSE* = 3, *p* = 0.830, $\eta_{p}^{2}$ < 0.01. No other effects were significant (*F*s < 2.285, *p*s > 0.146).

### Variability

**FIGURE 5:**
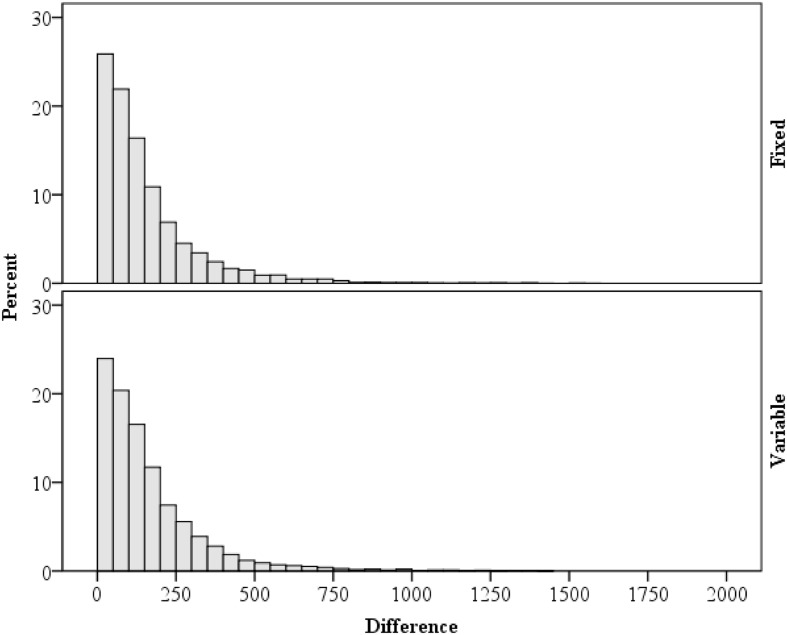
**Distribution of the differences in response times between the current and previous trial**.

As a supplementary analysis, variability in response times are considered. Of particular importance for rhythm setting is the relation between the current and immediately preceding trial (Schmidt, 2013b). Within each block, trials with a correct response on both the current and previous trials were analyzed. As predicted by the temporal learning account, response times on the previous trial were correlated with response times on the current trial, *r* = 0.20, *p* < 0.001 (Spearman’s ρ = 0.22, *p* < 0.001). Because of this, the stimulus–response interval (i.e., response time) was low in variability from trial to trial, with an average difference of ±159 ms. This mean is deceptively high, however, given the presence of RT outliers and the general heavy right skew (2.631), as illustrated in **Figure 5**. The median was ±110 ms. Note also that the distribution of difference scores are similar in the fixed and variable interval conditions. This contrasts sharply with the RSIs, which are either perfectly correlated with zero difference from trial to trial (fixed interval) or are perfectly uncorrelated (variable interval). These analyses indicate that, as intended, that stimulus–response intervals remain relatively unaffected by the RSI manipulation.

### Distribution Shape

**FIGURE 6:**
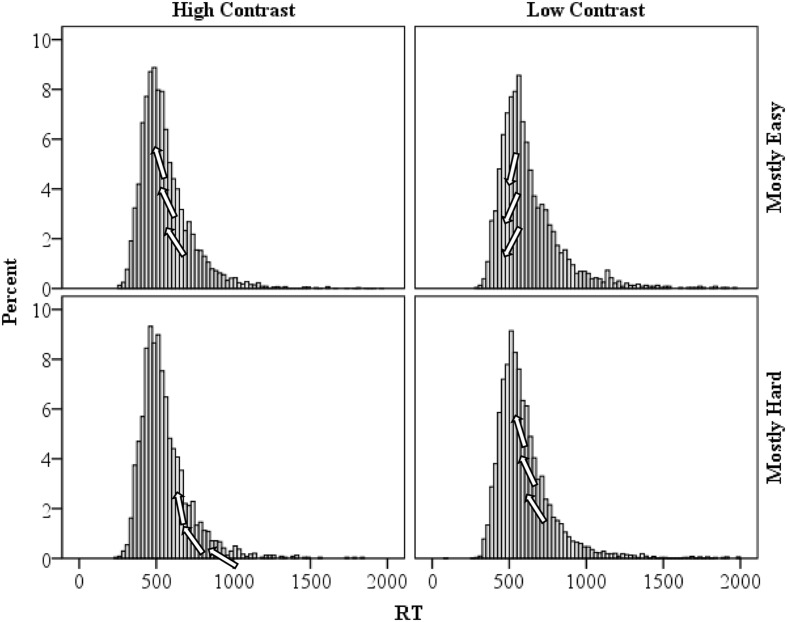
**Distribution of response times for high and low contrast items in the mostly easy and mostly hard conditions**. Arrows on the figures illustrate how the distribution is being affected by temporal learning.

As a final analysis, specific support for the temporal learning account of the proportion easy effect advocated in the present manuscript might be found in the distribution of response times. The distribution of response times for the high and low contrast items in the mostly easy and mostly hard conditions are presented in **Figure 6**. As a reviewer suggested, the faster rhythm in the mostly easy condition should result in a shifting of some of the relatively intermediate high contrast response times left (e.g., relative to the mostly hard condition). That is, relatively “average speed” high contrast trials will fit the mostly easy rhythm best and will therefore benefit the most from temporal expectancies. Speeding of these trials will increase the typical right skew observed in response times and also increase the peak of the distribution (i.e., leptokurtic or high kurtosis). For low contrast trials in the mostly easy condition, only the fastest of responses will be shifted left (i.e., benefit from the fast pace). Speeding of these trials will *reduce* the skew and peak (i.e., platykurtic or low kurtosis), relative to the mostly hard condition. In the mostly hard condition, it should be the reverse: relatively slow high contrast trials will benefit from the slower rhythm, reducing the skew and kurtosis; and relatively “average” low contrast trials will benefit, so skew and kurtosis will be increased (e.g., relative to the mostly easy condition). Consistent with this, high contrast trial skewness and kurtosis were higher in the mostly easy condition (skewness: 2.094, *SE*: 0.033; kurtosis: 8.148, *SE*: 0.066) than in the mostly hard condition (skewness: 2.041, *SE*: 0.050; kurtosis: 7.525, *SE*: 0.100). However, the bootstrapped 95% confidence intervals overlap for both skewness (1.830–2.330 vs. 1.689–2.385, respectively) and kurtosis (5.964–10.050 vs. 4.866–10.384), which might not be so surprising given the non-significant RT difference between mostly easy and mostly hard high contrast trials. Similarly, for low contrast items skewness and kurtosis were higher in the mostly hard condition (skewness: 2.395, *SE*: 0.033; kurtosis: 9.559, *SE*: 0.067) than in the mostly easy condition (skewness: 2.084, *SE*: 0.051; kurtosis: 6.628, *SE*: 0.102). In this case, bootstrapped 95% confidence intervals did *not* contain the opposing condition estimates for skewness (2.166–2.592 vs. 1.831–2.314) or kurtosis (7.814–11.040 vs. 4.992–8.163), indicating a statistically reliable difference in distribution shapes. These findings are thus consistent with the temporal learning view.

## Discussion

The present report provides valuable new insights into temporal expectancies and rhythmic responding in performance paradigms. As previously observed (e.g., Grosjean et al., 2001), overall response time was slowed when RSIs were variable. However, this overall slowing did not impair the proportion easy effect. In both the variable and fixed interval conditions, the effect was observed and at similar magnitudes. This, of course, is inconsistent with the response–response learning account discussed in the Introduction. Fixed RSIs are obviously more temporally regular than variable RSIs. As such, if participants were learning the intervals between one response and the next, then random variations of the RSI greatly impedes the possibility of learning a regular rhythm. In other words, it seems unlikely that one would observe a proportion easy effect in the variable RSI condition.

In contrast, the stimulus–response learning account proposes that the rhythmic biases responsible for the proportion easy effect are sensitive to the regularities between stimulus onset and the response. Although foreperiod duration (Sanders, 1966) and variability (Granjon et al., 1973; Requin et al., 1973) certainly affect response time on any given trial, a temporal regularity can still be acquired. As the present results demonstrated, the time between stimulus onset and a response obeys a sufficient regularity to promote a rhythm. This pace, of course, will be different with mostly easy relative to mostly hard items, which produces the proportion easy effect. It is prudent to point out, however, that the present experiment did not actually manipulate stimulus–response intervals. Of course, learning of the stimulus–response intervals seems the only plausible remaining account and, furthermore, these intervals (i.e., response times) are typically under the control of participants rather than the experimenter. That said, future research might aim to manipulate stimulus–response intervals directly, for instance, with filler items that have an enforced timing (e.g., a cued response window). It remains possible, for instance, that something entirely unrelated to rhythmic responding produces the proportion easy effect.

There are many different accounts of the mechanisms underlying rhythmic responding (Grice, 1968; Kohfeld, 1968; Ollman and Billington, 1972; Van Duren and Sanders, 1988; Strayer and Kramer, 1994a,b; Kinoshita et al., 2011; van Maanen et al., 2011). These accounts share many commonalities, but the present dialog was framed within a learning framework where participants use memories of response time durations to prepare for a response at an expected time. For instance, in the Parallel Episodic Processing model (Schmidt, 2013b, in press; Schmidt and Weissman, 2015), the model creates episodic memories that contain information about the stimuli presented, the response made, and, more critically for the present discussion, the response time. Recently encoded episodes are retrieved on each trial, and the stored response times can be used to anticipate responding at a particular time. More precisely, the response threshold (i.e., the amount of evidence required to select a response) is temporarily decreased at the time corresponding to the retrieved response times. As an example, if a string of high contrast stimuli are presented (e.g., in the mostly easy condition) and the response time to each of these is around 550 ms, then on the following trial the response threshold will temporarily decrease at around 550 ms. This will expedite responding to another high contrast trial. However, a low contrast trial is unlikely to benefit, because evidence for a response will simply be too low 550 ms into the trial to cross the temporarily decreased response threshold. However, after a string of low contrast trials (e.g., in the mostly hard condition), the response threshold will decrease later (e.g., 600 ms), which will tend to benefit low contrast trials more than high contrast trials. The distribution analyses in the present report add extra credence to this specific learning account: both skewness and kurtosis patterns were correctly predicted. Future work might aim to further distinguish between this learning account and some of the (highly related) alternatives (for a discussion, see Schmidt, 2013b). The current work helps to restrict the number of feasible accounts to those that propose no meaningful impact of variable RSIs and further suggest that learning of stimulus–response durations might be responsible for the effect.

It is notable that this observed pattern of results is somewhat different than what has been observed in the word reading literature, where *all* trials (easy or hard) are observed to be faster following easy trials (Taylor and Lupker, 2001). This suggests a more stable (rather than dynamic) adjustment of the response threshold. That is, after a (fast) easy trial the threshold is lowered and performance on any trial will be speeded. Of course, this specific timing account does not predict the observed results in the present report. Overall responding would have been globally faster in the mostly easy condition and, if anything, low contrast trials should have been responded to *faster* in the mostly easy condition (i.e., where the threshold for responding would be lower). Thus, the fixed threshold adjustment proposed by Taylor and Lupker (2001) fits well with the word reading data, but poorly with proportion easy (and proportion congruent) data. Similarly, the dynamic threshold adjustment account proposed in the current paper explains well proportion easy (and proportion congruent) data, but explains poorly the word reading data. However, the tasks in the two literatures vary in important ways (e.g., here a small set of repeated target stimuli are used, whereas a large set of novel stimuli are used in word reading research). It might therefore be proposed that the way in which participants adjust their response criterion depends on the task being performed. Future research might aim to investigate these issues further.

A secondary aim of the present report was to test whether random variations in RSIs might prove an effective means to eliminate rhythmic biases in investigations where rhythmic responding represents a confound. For instance, in attempting to determine whether conflict adaptation (or “conflict monitoring”) contributes to the proportion congruent effect, rhythmic response biases represent a confound (i.e., any effect might be due to rhythmic biases, conflict adaptation, or a combination of the two). Unfortunately, the present results indicate that variable RSIs do not control for rhythmic response biases. On the positive side, the present results do help to better understand the processes that might be producing rhythmic response biases. As such, the current results might provide hints for future research attempting to control for rhythmic biases. In particular, future research might aim to impair regularity in response times. Relatedly, manipulations aimed to equate response times in mostly congruent and mostly incongruent conditions (e.g., with fast or slow filler items) might prove especially useful in eliminating rhythmic biases in the proportion congruent effect.

As a final note, the relation between proportion easy and proportion congruent effects is worth considering. The two effects share obvious similarities. As such, evidence for temporal learning (or whatever other conflict-unrelated process produces the proportion easy effect) in the (conflict-free) proportion easy paradigm is informative for theorizing about proportion congruent effects. In particular, it seems likely that rhythmic response biases should, at least in part, contribute to the proportion congruent effect. The author has suggested elsewhere that there may be no proportion congruent effect independent of these simple learning biases (e.g., Schmidt, 2013a,b). Of course, the present results do not speak to this issue. Conflict was not manipulated in the current paradigm, so it remains entirely possible that *both* temporal learning and conflict adaptation contribute to the proportion congruent effect. In order to determine whether or not conflict adaptation does, indeed, play a role in the proportion congruent effect it will be necessary to determine a way to control for rhythmic response biases. Determining a way to achieve such a control is a worthwhile endeavor and the present investigation provides some initial steps forward.

## Author Contributions

The author confirms being the sole contributor of this work and approved it for publication.

## Conflict of Interest Statement

The author declares that the research was conducted in the absence of any commercial or financial relationships that could be construed as a potential conflict of interest.
